# A randomised controlled trial to evaluate the impact of indoor living space on dairy cow production, reproduction and behaviour

**DOI:** 10.1038/s41598-022-07826-9

**Published:** 2022-03-09

**Authors:** Jake S. Thompson, Christopher D. Hudson, Jonathan N. Huxley, Jasmeet Kaler, Robert S. Robinson, Kathryn J. Woad, Nicola Bollard, Jenny Gibbons, Martin J. Green

**Affiliations:** 1 School of Veterinary Medicine and Science, University of Nottingham, Sutton Bonington Campus, Leicestershire, LE12 5RD UK; 2 School of Veterinary Science, Massey University, Palmerston North, 4474 New Zealand; 3 Agriculture and Horticulture Development Board (AHDB), Stoneleigh, Kenilworth, CV8 2TL UK

**Keywords:** Reproductive biology, Risk factors, Epidemiology, Animal behaviour, Animal physiology

## Abstract

As a global society, we have a duty to provide suitable care and conditions for farmed livestock to protect animal welfare and ensure the sustainability of our food supply. The suitability and biological impacts of housing conditions for intensively farmed animals is a complex and emotive subject, yet poorly researched, meaning quantitative evidence to inform policy and legislation is lacking. Most dairy cows globally are housed for some duration during the year, largely when climatic conditions are unfavourable. However, the impact on biology, productivity and welfare of even the most basic housing requirement, the quantity of living space, remains unknown. We conducted a long-term (1-year), randomised controlled trial (CONSORT 10 guidelines) to investigate the impact of increased living space (6.5 m^2^ vs 3 m^2^ per animal) on critical aspects of cow biology, behaviour and productivity. Adult Holstein dairy cows (n = 150) were continuously and randomly allocated to a high or control living space group with all other aspects of housing remaining identical between groups. Compared to cows in the control living space group, cows with increased space produced more milk per 305d lactation (primiparous: 12,235 L vs 11,592 L, *P* < 0.01; multiparous: 14,746 L vs 14,644 L, *P* < 0.01) but took longer to become pregnant after calving (primiparous: 155 d vs 83 d, *P* = 0.025; multiparous: 133 d vs 109 d). In terms of behaviour, cows with more living space spent significantly more time in lying areas (65 min/d difference; high space group: 12.43 h/day, 95% CI = 11.70–13.29; control space group: 11.42 h/day, 95% CI = 10.73–12.12) and significantly less time in passageways (64 min/d), suggesting enhanced welfare when more space was provided. A key physiological difference between groups was that cows with more space spent longer ruminating each day. This is the first long term study in dairy cows to demonstrate that increased living space results in meaningful benefits in terms of productivity and behaviour and suggests that the interplay between farmed animals and their housed environment plays an important role in the concepts of welfare and sustainability of dairy farming.

## Introduction

A key challenge for global society is to negate the environmental impacts of agriculture, whilst ensuring a sufficient quantity of food can be delivered via socially acceptable methods^1^. Since livestock farming continues to be the largest user of global land cover^2^ and demand for livestock products is predicted to grow substantially worldwide^3^, the sustainability of livestock agriculture will remain of critical importance^4^. Recent decades have seen intensification of agriculture lead to increased food availability to meet continuing demands for animal products^3^, but a number of detrimental environmental effects have been described^5,6^. Solutions to enhance the sustainability of livestock farming must align with the three pillars of sustainability; to be socially acceptable for animal, farmer and society, to have a neutral or positive environmental impact and to allow economic reparation for continuation of development^7^. There is acceptance that environmental impacts could be limited through intensification in the right circumstances, that is by maintaining or increasing the same amount of food production from fewer animals^8–10^. However, it is also clear that variability of intensive farming management practices (examples include but are not limited to, husbandry and feeding practices) could influence animal health, welfare, productivity, land use and greenhouse gas (GHG) emissions^11^.

Technical developments in sustainable intensification have resulted in increased production efficiency alongside improvements to land utilisation, examples of which are livestock feed conversion^12,13^, genetics^14^ and precision livestock technologies^15^. However, the OIE (The World Organisation for Animal Health) have stated that production and animal welfare are inter-related^16^, and that sustainable intensification should encompass incentives for higher animal welfare standards as well as increased productivity and efficiency^17^.

The dairy industry provides a key nutritional source for many consumers worldwide^18^ but, in terms of sustainability, the management of dairy cows is complex. Generally, dairy cows in the Global North require periods of housing within their productive lifetime^19–21^, largely to protect them from seasonal adverse climatic conditions, for other management practices such as feeding consistent high energy rations and they live longer than most other food-producing animals. Although intensification of dairy farming has been shown to result in lower GHG emissions (such as CO_2_, N_2_0 and CH_4_) when based on equivalents per litre of milk produced^22,23^, the environment in which cows are housed is known to play a critical role in the magnitude of GHG production. An example of this is that different housing infrastructure, systems and configurations have been reported to influence emissions^24^. An appropriate housed environment is also considered critical for cow welfare, indeed it has been proposed that enhancing welfare through improved housing and husbandry can be more effective to promote health and productivity than therapeutic measures such as vaccination^25^. The conditions in which cows are housed are therefore fundamental to the sustainability of dairy farming and the welfare of farmed cattle.

An additional reason for research to be focused on dairy cow housing is that for society this topic is an emotive subject, especially when tied with the concepts of welfare and “naturalness”^26^. A number of studies have shown that naturalness, milk quality and cow health and welfare all ranked high in importance for consumers when making purchasing decisions^26–28^. It is increasingly important for farming practices to fit with societal and environmental goals, as well as providing suitable conditions for animal welfare^29–31^, in order for the industry to keep its societal licence to operate^32^. Understanding the fundamental impacts of housing conditions on animal productivity, behaviour and underlying physiological responses are essential for all livestock farming.

Despite the importance of dairy cow housing to the sustainability of the industry, quantitative research to evaluate and optimise housed conditions to achieve sustainable intensification in dairy farming is virtually non-existent. Consequently, there is a void of critical high quality evidence that key decision makers require to support national and transboundary objectives, policies and regulations^33^. The majority of dairy cows worldwide, particularly in the Global North, are housed for some time during the year with an increasing number of farms housing dairy cows year-round^20^ but even for the most fundamental requirement, the amount of living space required^34^, there are no compelling data to inform the debate. The current recommendations for freestall accommodation space allowances for dairy cattle worldwide have no scientific basis and show great variability; for example total space allowance recommendations range from 6.0 to 11 m^2^ per animal amongst countries worldwide^35–39^. For context an average adult commercial dairy cow would occupy in the region of 1.8–2 m^2^ when standing. Research relating how space allowance impacts livestock is lacking but in human literature, living space availability has been classed as the most important housing characteristic linked to health issues^40–42^. Further reports within the human literature highlight that living space is critical for health and wellbeing of occupants^43–45^, we hypothesise that living space would have similar benefits for housed dairy cows, given they are sentient beings and experiences within their environment are likely to impact their welfare.

We addressed this critical research gap by conducting a long-term experimental research trial to evaluate the impact of living space on dairy cow productivity, reproductive biology and behaviour. Our results show that increased living space in dairy cow accommodation leads to increased milk production, a deterioration in reproductive performance and altered cow behaviours likely to be positive for welfare. Overall the results strongly suggest that increased living space is beneficial for dairy cows and could therefore enhance the sustainability of dairy industry and welfare of animals worldwide.

## Results

### Overview of experimental design

The study aim was to quantify the effect of a spatial intervention on milk production and reproductive performance of housed lactating dairy cows and to assess the impact of changes to the housed environment on cow location time budgets. We undertook a long term (364 day) randomised controlled trial to evaluate space allowance; the ‘control space’ group had 9 m^2^ total space (including 3 m^2^ of living space, a bespoke previously defined area of the space within dairy cow accommodation which indicates space greater than that considered a baseline requirement^34^) per cow and the ‘high space’ group had 14 m^2^ total space (including 6.5 m^2^ of living space) per cow. All other aspects of the environment, management and animal husbandry remained identical between groups.

### Additional living space results in increased milk volume but is dependent on cow maturity

Observational studies have provided evidence for how the housed environment could impact dairy cow milk production^46^, but the impact of living space on milk yield within a carefully controlled environment has not been investigated.

**Figure 1:**
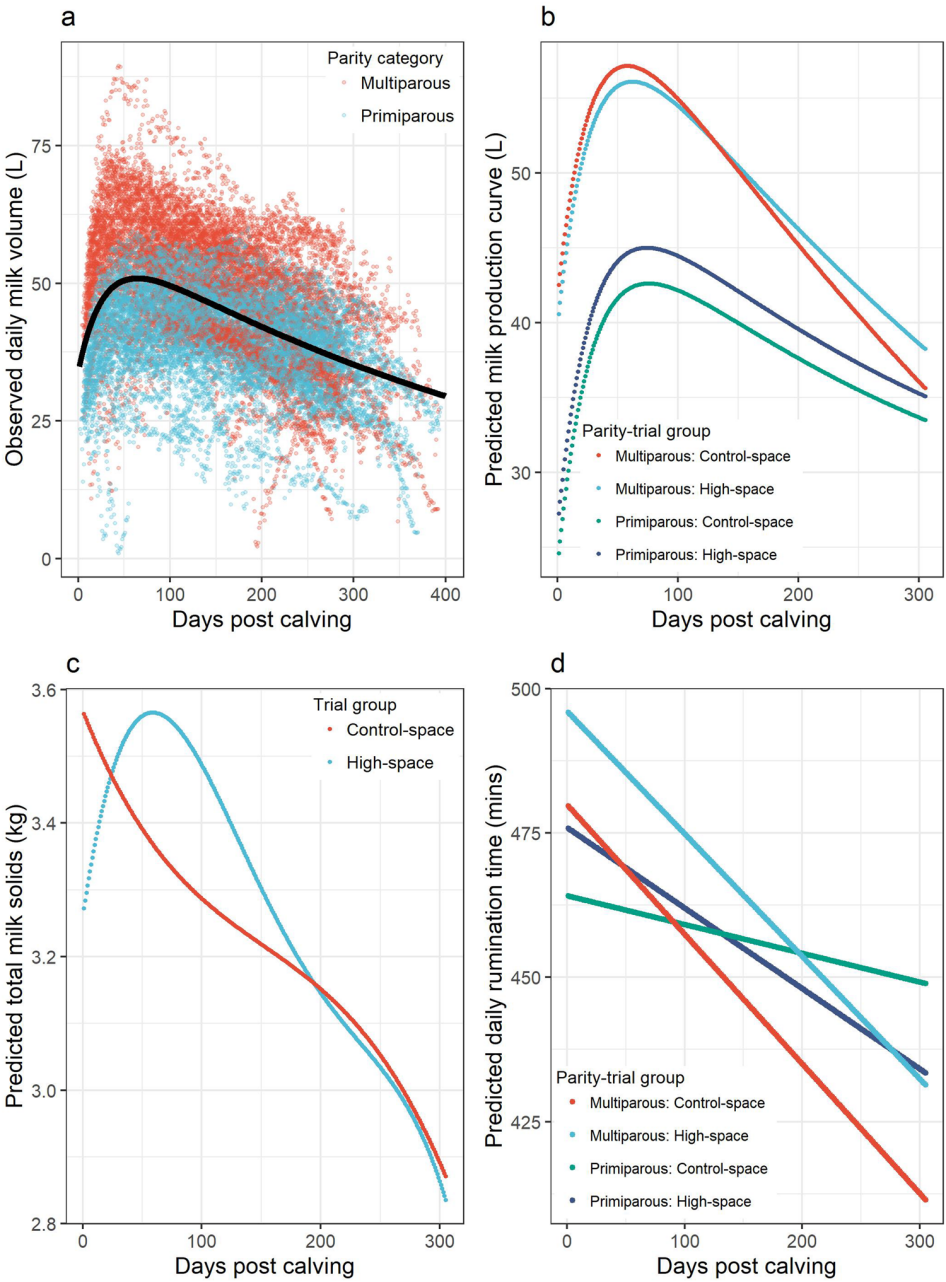
Plots to illustrate the results of the production analysis, based on milk volume, milk solid and rumination data, for a randomised controlled trial assessing the impact of living space on housed dairy cows. (**a**) Scatterplot of daily milk yields in litres (y-axis) by days in milk (x-axis) for all cows (n = 150) throughout the randomised controlled trial period. The black line shows the non-linear model regression lactation curve prediction for all cows on trial. (**b**) Dot-plot depicting the predicted 305-day lactation curves using the parameters of the daily milk volume residual mixed effects model (Eq. 2) for the four groups of cows investigated: Primiparous—High-space (light blue); Primiparous—Control-space (green); Multiparous—High-space (red); Multiparous—Control-space (dark blue). (**c**) Plot to illustrate the predicted milk constituent curves from the mixed effects model parameters (Eq. 3) for the high (blue) and control (red) space groups. (**d**) Plot to illustrate the predicted rumination time using the mixed effects model estimates (Eq. 4) in minutes per day (y-axis) by days in milk (x-axis) for the four parity-trial groups investigated: Primiparous high-space (light blue); Primiparous control-space (green); Multiparous high-space (red); Multiparous control-space (dark blue).

A two-step modelling method was used to assess the differences in milk yield between groups. Parameter estimates from a non-linear model fit to daily milk volume data (independent of trial group and parity) to determine the relationship between daily milk volume production and days in milk, as described by Ehrlich^47^, were scale = 60.19 (t value = 139.1), ramp = 30.2 (t value = 17.9), offset = − 4.33 (t value =  −4.3) and decay = 0.001782 (t value = 52.1). The predicted regression curves alongside observed data are illustrated in Fig. 1a.

In step two, residuals from the non-linear model were calculated for each day after calving (each residual represented the difference between observed and predicted milk yield for each cow) and residual milk yield values were used as the dependent variable in a mixed-effects model to evaluate the effect of living space on milk yield. Results are provided in Table 1.

**Table 1:** Table of mixed effects model estimates for prediction of the daily milk volume residuals calculated from the non-linear model, using days in milk (DIM), parity and trial group (with interactions) as covariates, using data from a long-term randomised controlled trial to evaluate the impact of living space on dairy cow productivity, reproductive biology and welfare.

Model outcome: daily milk yield residual (L)			
Fixed effects	Estimates	CI	*P*
(Intercept)	− 10.31	− 12.88 to − 7.75	
DIM	0.03	0.03 to 0.03	< **0.001**
Primiparous—control space	*(reference)*		
Primiparous—high space	2.64	− 0.98 to 6.27	0.153
Primiparous—high space * DIM	0.00	− 0.01 to − 0.00	**0.002**
Multiparous—high space	16.01	12.36 to 19.66	< **0.001**
Multiparous—high space * DIM	− 0.04	− 0.04 to − 0.03	< **0.001**
Multiparous—control space	17.96	14.32 to 21.61	< **0.001**
Multiparous—control space * DIM	− 0.05	− 0.05 to − 0.05	< **0.001**
**Random effects**			
Residual variance	16.37		
Random effect variance	64.23_cow_parity_		
Intraclass correlation	0.8		
Number of random effect groups	150_cow_parity_		
Number of observations	25,594		
Marginal R^2^/conditional R^2^	0.233/0.844		

Significant values are in [bold].

Cows in the high space group produced significantly more milk than those in the control space group and the impact of living space on milk yield was conditional on cow parity and days in milk. For visualisation, model results are illustrated in Fig. 1b. Model estimates were combined with the non-linear model residuals for each corresponding day in milk integer value to produce 305-day milk yield predictions for each trial-parity group; primiparous cows in the high space group produced 12,235L per 305 day lactation compared to 11,592L for primiparous cows in the control space (643L difference) and multiparous cows in the high space produced 14,746L as opposed to 14,644L for multiparous control space cows (102L difference). The fit of the final mixed effect model was deemed to be good.

### Additional living space results in increased milk constituents and rumination times

Depending on the payment structure of a farms milk contract, milk volume payments may also be dependent on milk constituent percentages (e.g. fat and protein). Therefore difference in milk volume between trial groups could have been linked to dilution of the milk constituents, fat and protein^48,49^, which could negatively affect milk value. Milk fat and protein production (total milk constituents in kilograms) were evaluated for all cows using mixed effects models (Table 2). Five cows were not included in the final mixed effects model due to missing data (failure of sampling or laboratory procedure). The resulting pattern was similar to that for milk volume with highest levels of milk constituents produced at peak volume of production (Fig. 1c). Cows in the high space group produced substantially more milk constituents (968.2 kg) than those in the control group (951.9 kg) over a 305-day period. Analyses of model residuals indicated fit was good.

**Table 2:** Results from the mixed effects model to predict daily milk constituent production (kg) from monthly milk recording samples using trial group and days in milk (DIM) using monthly milk recording data from a year-long randomised controlled trial to evaluate the impact of living space on dairy cow productivity.

Model outcome: daily total milk solid production (kg)			
Fixed effects	Estimates	CI	*P*
(Intercept)	3.26	2.93 to 3.60	
High space	*(reference)*		
Control space	0.31	− 0.16 to 0.78	0.198
DIM	0.012	0.001 to 0.023	**0.032**
DIM^2^	− 1.5 × 10^−4^	− 2.7 × 10^−4^ to − 2.6 × 10^−5^	**0.017**
DIM^3^	5.9 × 10^−7^	7.7 × 10^−8^ to 1.1 × 10^−6^	**0.024**
DIM^4^	− 8 × 10^−10^	− 1.5 × 10^−9^ to − 1 × 10^−10^	**0.027**
Control space * DIM	− 0.017	− 0.032 to − 0.001	**0.037**
Control space * DIM^2^	1.7 × 10^−4^	− 2.1 × 10^−6^ to 3.5 × 10^−4^	0.053
Control space * DIM^3^	− 6.5 × 10^−7^	− 1.4 × 10^−6^ to 8.4 × 10^−8^	0.083
Control space * DIM^4^	8 × 10^−10^	− 2 × 10^−10^ to 1.8 × 10^−9^	0.112
**Random effects**			
Residual variance	0.26		
Random effect variance	0.33		
Intraclass correlation	0.56		
Number of random effect groups	145		
Number of observations	788		
Marginal R^2^/conditional R^2^	0.076/0.593		

Significant values are in [bold].

Rumination in cattle is a key physiological mechanism for the digestion of cellulose and increased rumination time has been linked to increased milk volume in dairy cows^50^. The mixed effects model revealed that daily rumination times were different between trial-parity groups with significant interactions between trial-parity group and days in milk (Table 3). To illustrate these effects, model predictions are displayed graphically in Fig. 1d. For multiparous cows, mean daily rumination times were consistently higher for those in the high space group (mean 462 min/day during a 305-day lactation) compared to those in the control group (444 min/day). For primiparous cows, the mean rumination times for each group differed according to stage of lactation with cows in the high space group having increased rumination times until approximately 130 days into lactation at which time the effect was reversed (Fig. 1). Notably, in the first 100 days in milk, when cows moved from the commencement of a lactation to peak yield, the predicted mean rumination times per group were; high space primiparous cows 469 min/day, control primiparous cows 462 min/day, high space multiparous cows 485 min/day and control space multiparous cows: 469 min/day.

**Table 3:** Results from the mixed effects model (Eq. 4) to predict daily rumination time (minutes) from parity-trial group and days in milk (DIM) using data from a long-term randomised controlled trial to evaluate the impact of living space on housed dairy cows.

Model outcome: daily rumination time (min)			
Fixed effects	Estimates	CI	*P*
(Intercept)	462.9	449.2 to 476.7	
DIM	− 0.039	− 0.056 to − 0.022	< **0.001**
Primiparous, control space	*(reference)*		
Primiparous, high space	13.05	− 6.44 to 32.53	0.189
Multiparous, control space	16.08	− 3.53 to 35.69	0.108
Multiparous, high space	32.26	12.65 to 51.87	**0.001**
DIM * Primiparous, high space	− 0.10	− 0.13 to − 0.08	< **0.001**
DIM * Multiparous, control space	− 0.18	− 0.20 to − 0.15	< **0.001**
DIM * Multiparous, high space	− 0.17	− 0.19 to − 0.14	< **0.001**

Significant values are in [bold].

### Cows with more living space take longer to conceive

**Figure 2:**
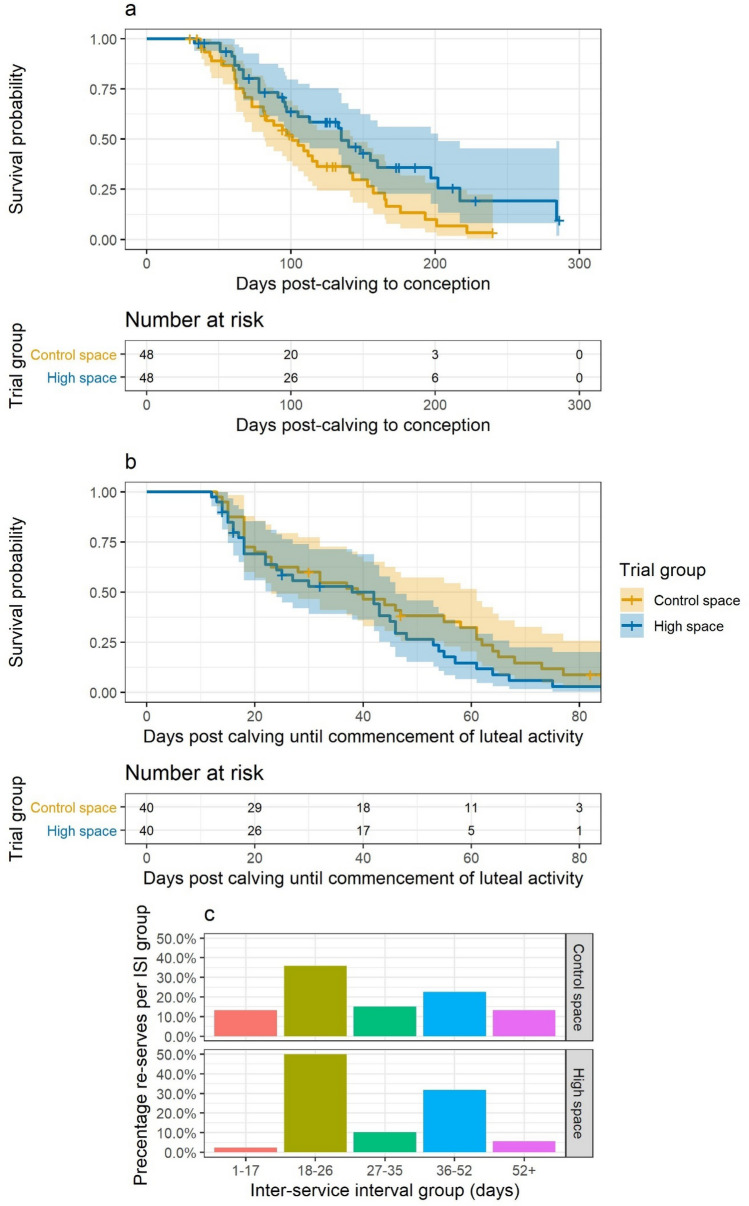
Graphs based on fertility data analysed for a long-term randomised controlled trial which evaluate the impact of living space on housed dairy cows. (**a**) Results of time to conception analysis for matched cows that entered the trial non-pregnant (n = 48 pairs). Top: Kaplan–Meier plot of survival to conception for the high space (blue) and control space (yellow) trial groups. Respective colour shading represents the 95% confidence interval. Bottom: Risk table showing the associated number of cows non-pregnant at 0-, 100-, 200- and 300-days in milk (DIM) for each trial group. (**b**) Results of time to commencement of luteal activity (CLA) analysis (n = 40 pairs); colour shading represents 95% confidence interval as for (**a**). (**c**) Bar charts of inter-service intervals (ISI) by trial group (Top: control space; Bottom: high space). A higher percentage in the 18–26 day period (yellow bars) indicates normal cow cyclicity and provides a measure of the sensitivity of oestrous detection.

Reproductive efficiency is an important trait in dairy cows and has become a key selection criteria following a historical focus on milk volume in most genetic selection indexes^51^. Our underlying hypothesis was that increased space would improve fertility and reduce the time to conception period. This fundamental timepoint drives herd economic sustainability^52^, longevity in the herd^53^, and is considered the main indicator of reproductive performance^54^. Results of the time to event analysis^55,56^ undertaken on the 48 pairs of non-pregnant cows at trial entry, are provided in Table 4 and illustrated in Fig. 2. Fit of the survival models were found to be good and model assumptions were met.

**Table 4:** Parameter estimates from the Cox proportional hazards model using data from cow-pairs using data from a year-long randomised controlled trial to evaluate the impact of living space on dairy cows that were non-pregnant at trial entry by trial-parity group.

Term	Estimate	Std error	Statistic	*P* value	Lower CI	Higher CI
Primiparous control space	*(reference)*					
Primiparous high space	0.48	0.35	− 2.09	**0.036**	0.24	0.95
Multiparous high space	0.58	0.36	− 1.52	0.13	0.29	1.17
Multiparous control space	0.84	0.33	− 0.54	0.59	0.44	1.60

Significant values are in [bold].

The hazard of pregnancy was lower in the high space compared to control group (hazard ratio = 0.58, *P* = 0.03) which resulted in a median time from calving to conception of 135 days for cows in the high space group and 101 days for cows in control group. However, the difference in time to conception was greater for primiparous cows; the high space primiparous cows median time to conception was 155 days and the control primiparous cows was 83 days (*P* = 0.04). For multiparous cows, in the high space group the time to conception was 133 days (*P* = 0.13) and in the control group it was 109 days (*P* = 0.59).

To further explore time to conception between trial groups, a subset of cows that entered the trial at < 14DIM were evaluated. Similar patterns were observed in these data; the hazard of pregnancy continued to be significantly lower in the high space compared to control group. Compared to the reference group of primiparous cows in the control group which had a median time to conception of 83 days in milk, the median time to conception for the high space primiparous cows was 202 days (hazard ratio = 0.43, *P* = 0.052) and multiparous high space cows was 113 days (hazard ratio = 0.60, *P* = 0.21). The multiparous control space cows had a median time to conception of 101 days in milk and were shown to be perform similarly to the primiparous cows in this group (hazard ratio = 1.00, *P* = 0.99).

### Time to conception is not related to underlying reproductive physiological parameters

Further detailed analyses of reproductive data and physiology were conducted. Analysis of the artificial insemination (service) data revealed that cows in the high space group were served more times, 120 versus 99, but this resulted in fewer pregnancies, 29 versus 37 (Chi-squared test, *P* = 0.049) than the control space group. A normal interservice interval (ISI) corresponds to the normal oestrous cycle of the cow which is widely considered to be 21 days but can be 22 days for modern dairy cows with normal intervals ranging between 18 and 26 days^57^. The distribution of ISIs between trial groups are shown in Fig. 2c and illustrates that the distribution of intervals appeared superior for the high space group cows. This implies that oestrus was being displayed and detected at least as efficiently in the high space group compared to the control and that the key physiological difference between trial groups was in the likelihood of conception when a service occurred rather than oestrus cyclicity.

Progesterone levels in milk were used to investigate the underlying reproductive physiology between trial groups, such as commencement of luteal activity and normality of oestrous cyclicity; as these have been linked to reproductive success^58,59^. The number of each profile-type was similar for both trial groups as follows; high space: 8 Normal, 12 Abnormal, 19 ‘Delayed ovulation’ (DOV); Control-space: 11 Normal, 11 Abnormal, 17 DOV, *P* = 0.73^58^. The time-to-conception analysis was re-run with the addition of progesterone profile as a potential confounding variable, but no difference was identified in the impact of trial group on time to conception.

Commencement of luteal activity was calculated from the progesterone profiles as an indicator of reproductive health^60^ and comparisons made between trial groups. Minimal difference was identified between groups. Median time for the commencement of luteal activity after calving for cows in the high space was 38 days (95% CI: lower = 24, upper = 46) and for the control group was 39 days (95% CI: lower = 23, upper = 61), as shown in Fig. 2b.

Anti-Mullerian hormone (AMH) samples from cows between 28-35DIM were analysed to provide an indicator of post-calving ovarian follicle development and possible subsequent fertility performance^61^, but there was no difference between groups; the high space group had a median AMH concentration of 122 (interquartile range: 73.5–271) and the control group had a median of 150 (interquartile range: 98–254.5), *P* = 0.40.

There were no significant differences between trial groups in presence of endometritis (*P* = 0.58), uterine measurements (*P* = 0.51), maximum antral follicle count (*P* = 0.85) or presence of a corpus luteum by 42 DIM (*P* = 1.00), these were found to be similar between groups throughout the trial.

Negative energy balance was assessed through measurement of serum beta-hydroxybutyrates (BHB) and non-esterified fatty acids (NEFA) from blood samples taken in weeks 2, 3 and 4 post-partum. The prevalence of abnormal readings for both parameters was low and not significantly different between the trial groups. When tested as a confounding variable, neither the concentration of BHB or NEFA were found to alter parameters in the final time to conception survival models; no confounding effect was detected.

### Associations between milk volume and reproductive performance

#### The difference in milk volume between intervention groups is not explained by differences in times to conception

**Figure 3:**
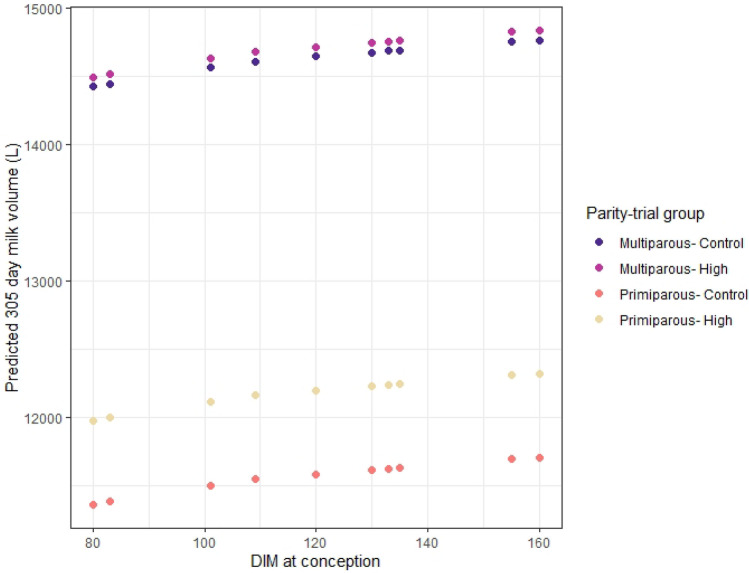
Scatterplot to illustrate the predicted 305-day milk yields (y-axis) by days in milk (DIM) at which a cow conceived (x-axis) based on data collected from a long-term randomised controlled trial to assess the impact of living space on housed dairy cows. The predictions are grouped by parity-trial group.

Energy is required to support a pregnancy^62^, therefore “number of days pregnant” (DP) was included as a predictor variable to the milk volume mixed-effects model to explore the hypothesis that the difference in 305-day milk predictions between the high space and control group was caused by the difference in observed reproductive performances. Model results revealed that in early pregnancy (up to 100 DP) there was no effect of pregnancy on milk volume, but as pregnancy progressed milk volume decreased such that by 201–250 DP this accounted for a reduction of 6.7L per cow per day (*P* < 0.001). When this model was used to predict milk volume per cow over a 305-day lactation (Fig. 3), it was identified that for primiparous animals the 72 day difference in median days to conception was associated with a 28L difference in milk yield per lactation. Therefore, of the total predicted difference of 645L for primiparous cows over a 305-day lactation, 28L could be attributed to a difference in reproductive performance, the additional 615L being associated with other effects of increased living space allowance. A similar calculation for multiparous cows identified that an increase of 35L in milk volume per cow per 305-day lactation could be attributed to differences in reproductive performance and therefore that 69L per lactation were associated with other effects of increased living space allowance (the full effects of different reproductive scenarios can be viewed in Fig. 3).

#### Milk production prior to service did not affect the outcome of insemination

Since cows in the high space group produced higher milk volumes but had poorer conception rates, it was hypothesised that there could be a causal influence of milk yield on conception. A binomial logistic regression model was employed to evaluate the probability of conception at each service which included a predictor variable for the 7-day milk volume prior to that service. Other predictors were days in milk and trial group. No effect of milk volume was identified (odds ratio = 1.00. *P* = 0.87), which indicated that increased milk volume did not influence the probability of conception. The effect of group allocation remained significant, with the odds of conception at a service being increased in the control space group (odds ratio = 2.68, *P* = 0.026).

#### Cows with more space had altered behaviours; increased time in lying areas and decreased time spent in passageways

**Figure 4:**
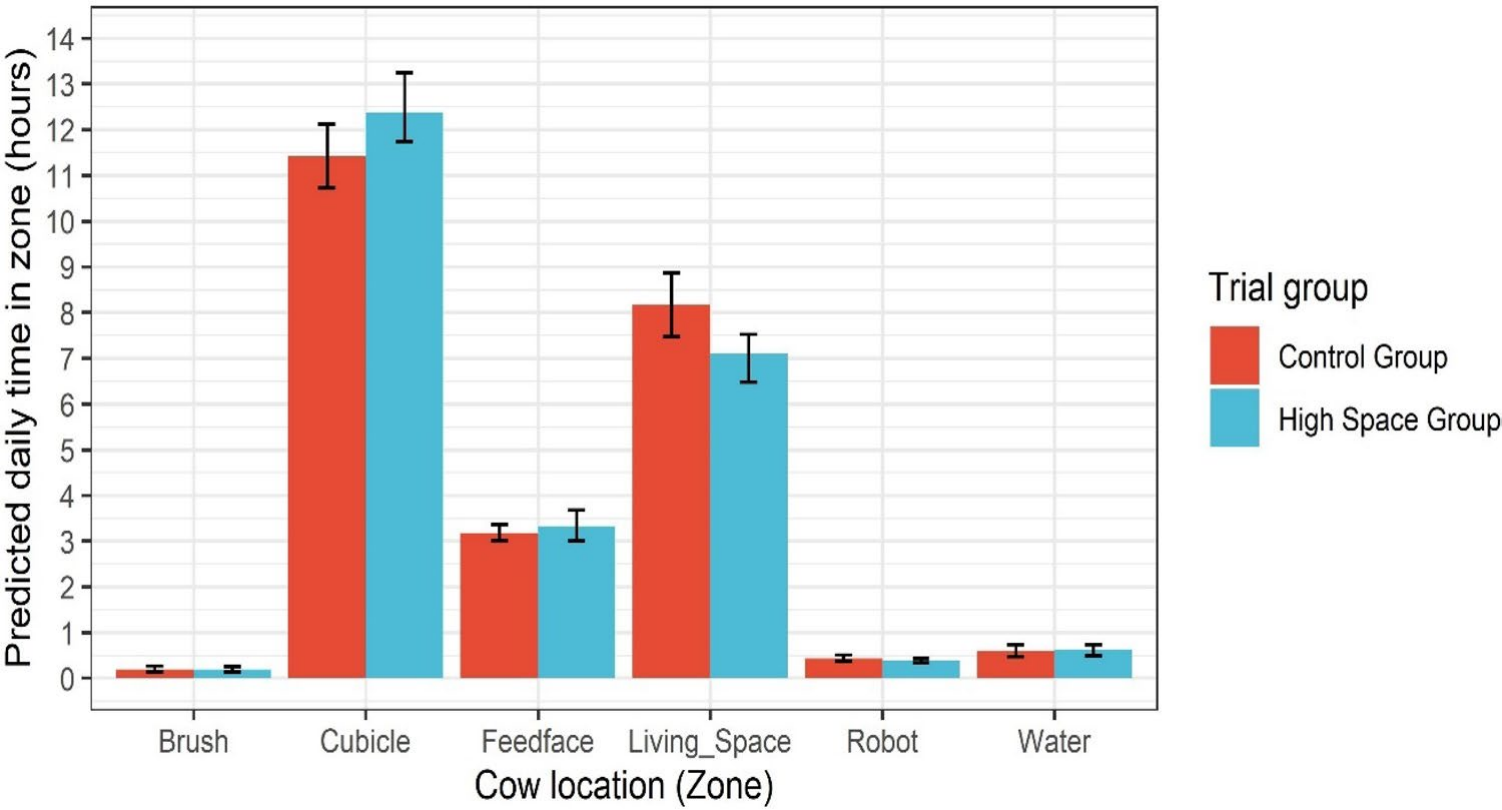
Plot of the final results of the time budgets for cows in each trial group during the sampling period. Cows in the high space group (red) spent significantly more time in cubicles and less time in living space areas in comparison to cows in the control space group (blue). The error bars (black lines) represent the 95% confidence interval of the mean for each location. Cubicle and living space areas show statistically significant differences between trial groups. These graphs are based on location data from a long-term randomised controlled trial which evaluated the impact of living space on the behaviour of housed dairy cows.

The welfare of farmed animals, linked with their behaviour^63,64^, is a topic which is emotive^65^ and discussed with increasing frequency by the industry, as well as by consumers and wider society. Results of the daily time budgets for key area zones for 16 matched cow pairs, measured using an electronic geolocations system, are illustrated in Fig. 4; significant differences were identified in two behaviours. Cows in the high space group (12.43 h/day, 95% CI = 11.70–13.29) spent significantly more time in lying areas, 1.01 h (61 min) per day longer, than cows in the control group (11.42 h/day, 95% CI = 10.73–12.12). Cows in the control group spent 1.07 h (64 min) per day longer in the living space areas (including passageways and loafing areas; 8.17 h/day, 95% CI: 7.48–8.88) than the high space group (7.10 h/day, 95% CI: 6.48–7.53). Times spent on other activities, feeding, drinking, use of a mechanical brush and milking were similar between groups.

Daily time budget analyses using different subsets of data (selected using different data quality criteria) or altered bootstrap methodologies, gave similar results to those described above and are therefore not presented.

## Discussion

This long-term randomised controlled trial identified that by changing the most basic attribute of the dairy cow housed environment, living space, fundamental differences in rumination, productivity, behaviour and reproduction occurred. It was notable in our results that the availability of additional living space appeared to affect some biological processes but not others. The key output of dairy farming, litres of milk (and mass of milk solids) produced per cow, was seen to increase; the effect was especially marked in primiparous animals. These observed differences are relatively small in comparison to the overall individual production of these animals, however when scaled to a herd level, the differences would provide a substantial financial income for a single change in management practice, which would continue each year thereafter. This increase in milk production appeared to be underpinned by increased lying and rumination times, markers of an efficient digestive process^50,66,67^. The particular response in milk output of younger animals suggests an additional behavioural component may also be involved; the additional space possibly allowing younger cows to exist with fewer antagonistic interactions with older, more dominant herd mates^68–71^ which may be linked with the enhanced milk production observed^46,72^. Our behavioural data supports this hypothesis since cows with more living space spent less time in passageway areas, the areas where negative cow to cow interactions are most likely to occur^73,74^. Although no prospective controlled trials have been conducted previously to evaluate the impact of additional living space for dairy cows, a similar association between milk production and space allowance has previously been described for primiparous animals in an observational study of 204 herds^46^. This previous study reported that milk production was 28.84L greater per square metre of space allocation per lactation for primiparous animals and less in multiparous animals, ranging from 7.61 to − 8.95 L per m^2^ per lactation, dependent on parity. Interestingly, these reported effect sizes were much smaller than those we identified in this study. Given it is difficult to evaluate causality and to establish unbiased effects in observational research^75,76^, this randomised controlled trial provides clear evidence of a causal relationship between milk yield and space allowance as well as providing a robust estimate of the likely effect size.

Interestingly, some notable biological processes were not altered by additional living space and these included those influencing reproductive physiology such as progesterone profiles, anti-Mullerian hormone and ultrasonic detection of ovarian follicles. Despite similarity in hormonal profiles, overall reproductive performance differed between trial groups, with the unexpected result that cows with increased living space took longer to become pregnant. This reduction in fertility performance was not negatively associated with milk yield since cows with higher yields tended to conceive soonest. A possible explanation for the improved reproductive performance in cows with less living space comes from the suggestion that short term stress can, in some circumstances lead to heightened reproductive performance^77,78^. However, the underlying mechanisms that underpin this difference in fertility could not be ascertained in this study and remain unknown. Given that chronic stress has generally been reported to reduce reproductive performance in dairy cattle^79,80^, the unforeseen patterns of fertility identified in this research require detailed future investigation.

The magnitude of difference in daily time budgets between the high and control space groups (1 h per day in lying areas and one hour per day in passageway areas) is indicative of a large and important effect of living space on behavioural expression. Very few previous studies have been conducted to evaluate associations between time spent in lying areas and passageways and characteristics of a cow’s housed environment, and none over a prolonged period. A previous experimental study reported that lying times increased (by ~ 2%, equating to approximately 30 min per day) as pen size increased and stocking densities were reduced^69^. The study, however, was conducted over relatively short (7-day) experimental periods, making the results difficult to interpret in terms of how cows would respond or adapt over longer periods of time. Another experimental study, using thirty cows from a single herd split into six groups, reported that lying times increased by 6.3 h per day, as floor space allowance increased from 3.0 m^2^ per cow to 10.5 m^2^ per cow^81^. This research was conducted using a relatively uncommon dairy system (a rubber matted area from which cows did not have access to pasture), and the differences in space allowance between treatment groups were extreme. Consequently, it is difficult to directly compare this study to the current research although the principle of increased space leading to increased time in lying areas and less time idling in passageways appears to hold. Nonetheless, our research and these previous studies highlight a similar general trend and provide firm evidence that living space is of fundamental importance for housed dairy cows. The influence of living space on cow behaviour is further supported by research that suggests that subordinate cows utilise excess space (such as loafing areas) in times of increased competition (e.g. feeding) to reduce the likelihood of an agonistic interaction occurring^82^. Our controlled trial, however, clearly demonstrates over a prolonged period, that increased living space leads to fundamental changes in dairy cow behaviour and specifically to increased time in lying areas rather than idling in passageways.

The space to exhibit normal behaviours is commonly cited as an important consideration for the welfare of dairy cows during housing^83–86^. Increased lying times are generally considered an indicator of enhanced welfare^87–89^ but lying times are confounded many other variables which makes them difficult to interpret^90^. However, a potentially more important measure could be time spent in the passageways/living areas, which is thought to represent a difficulty of free movement to access more valuable resources when less space is available^68,69,73,74^. This is important to distinguish because such space will allow cows uninhibited access to the areas from which they benefit (e.g. lying areas, feeding areas, environmental enrichment) and this may also help illustrate to consumers and citizens that cows are able to move freely. Therefore the behaviour changes associated with additional living space identified in this research are likely to represent enhanced welfare for these cattle and could be viewed positively by consumers and citizens who deem the welfare of housed cows an important issue^9,26^. Indeed, beyond improvements to productivity and financial returns, for dairy farming to promote positive societal perceptions of the industry and to continue to operate, it needs to address the expectations of the general public concerning the ethical and moral treatment of dairy cows^91^. Increased living space may be one route to address such concerns.

There are limitations of this study that should be considered. Whilst the research demonstrated important trends for the impact of space allowance on housed dairy cows, the results are a comparison between two specific scenarios, thus the findings should be generalised to other situations with caution. This highlights the need for further research on living space allowances; for example, additional scenarios to be examined could include further increases in living space availability, the impact of different durations of the housed period and the relevance of breed differences. However, our results provide strong and unique evidence that living space fundamentally impacts the biology of dairy cows and highlights the importance of this type of research to inform global animal health and welfare debates.

Due to its nature, full blinding of researchers was not possible during the study; measurements that could not be blinded included endometritis scores and reproductive organ scanning. To mitigate this, it was decided that the majority of key outcome data were gathered from laboratory analysis, sensor data and other objective data (e.g. date of conception) to help remove unconscious bias. In addition, to further mitigate potential bias, for data analyses the researchers were blinded to cow identification number and trial group such that analyses were conducted without this knowledge.

A further limitation was identified regarding aspects of the statistical analyses undertaken which could have been impacted by the available sample size of the data based on ethical approval restrictions. The sample size calculations for the randomised controlled trial were set to investigate all cows on trial irrespective of characteristics (e.g. parity). Therefore, statistical power was reduced when smaller sub-groups of data were used, such as dividing cows into parity group. Despite this, however, the effect sizes between groups were often of sufficient size to detect significant differences between subset groups, but it is possible some smaller effects were not discovered.

Finally, it should be noted that the trial groups contained a higher proportion of primiparous animals than found on some commercial farms. It is unclear whether, or the extent to which, results may change with different parity structures present on farm, this would be a worthy subject for additional research.

As a global society, we have a duty to provide suitable care and conditions for farmed animals yet in many circumstances, and particularly for the housed environment, evidence is minimal or absent. Among many current topics, for modern farming methods to remain socially acceptable and sustainable^92^, a greater understanding of the impact of an animal’s housed environment is needed; there is a need for substantial underpinning science of the extent to which animal behaviour, reproduction and productivity may be affected by living environment. Additionally, citizen perceptions of farm animal living conditions are highly polarised^26^ and the outcomes of any changes demanded may be conflicting. Therefore, high quality, quantitative evidence is urgently required to reveal how animals are affected, both positively and negatively, by the indoor conditions in which they are kept. Without such evidence, farmers, citizens, consumers, national and international decision makers are left devoid of scientific data regarding management practices, and thus policies and regulations are subject solely to opinion rather than appropriate scientific evidence.

The need for efficient food production has never been greater^93^ and key areas to increase efficiency of dairy farming have been suggested as: feeding less human food, sourcing regionally appropriate animals, improved health and nutrition^93^, and using technological advances^94^. The impact of the precise conditions under which animals are kept have been and remain continually overlooked. This study demonstrates that, given additional living space at housing, dairy cows will increase milk output, even in already very high yielding animals, and fewer animals would therefore be required per litre of milk produced to feed the human population. The results illustrate that the interplay between farmed animals and their housed environment has the potential to enhance both productivity and welfare and hence the sustainability and public acceptance of farming. Having been continually overlooked, it is time for greater attention to be paid to living space availability for farmed animals.

To conclude, this long-term randomised controlled trial, which assessed two living space scenarios for housed dairy cows, demonstrated that additional living space for dairy cows resulted in increased milk production, changes to reproductive performance and increased time cows spent in lying areas.

## Methods

### Permissions

Ethical permission was granted by the Home Office and the University of Nottingham ethical review committee; the study was conducted under the Animal [Scientific Procedures] Act 1986, license number MG_P07992717. The study adhered to all aspects of the CONSORT 10^95^ and ARRIVE^96^ guidelines to inform both study design and reporting. Methods were undertaken in accordance with Home Office guidelines and regulations.

The research comprised a randomised, controlled [1:1], long-term (1 year), longitudinal, parallel-group (pairs of adult dairy cows matched by parity and days in milk), cross-over (group location within facility) study to evaluate superiority/inferiority of a space allowance intervention for housed dairy cattle.

### The research environment

**Figure 5:**
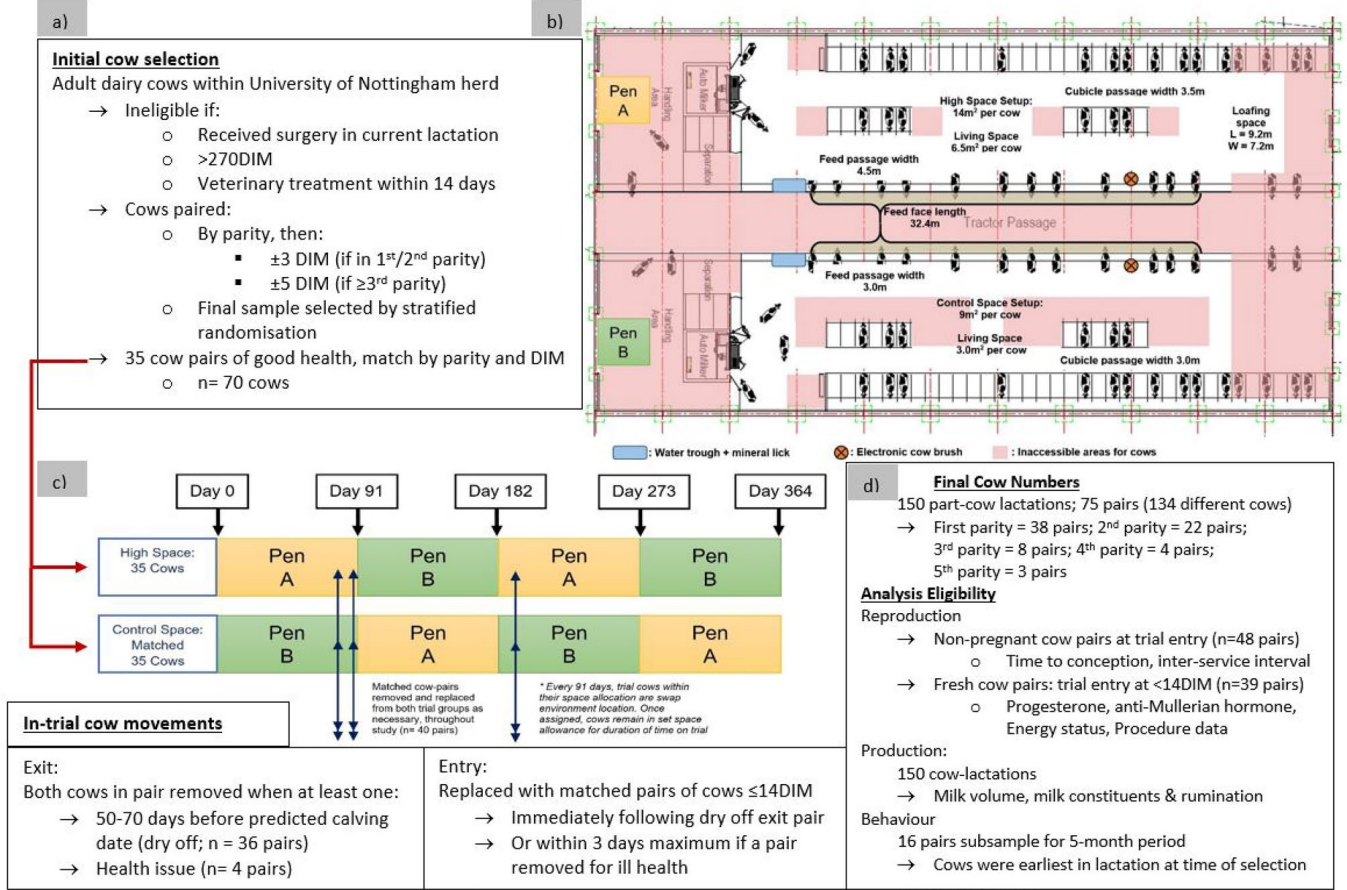
Schematic of a long-term randomised controlled trial study design to evaluate the impact of living space on housed dairy cows, including: (**a**) cow-selection (DIM: days in milk)); (**b**) cubicle shed floorplan with associated space comparisons (red areas inaccessible to cows during trial conditions); (**c**) Cross-over schematic detail cows entry and exit; (**d**) sample size and analysis detail.

The study took place over 364 consecutive days starting on 16th July 2018 until 15th July 2019 using a single, novel, state-of-the-art freestall dairy cow housing facility at The University of Nottingham, UK. The research building contained two pens that were a precise mirror image but that allowed modification of the internal layout (Fig. 5). The experimental intervention was a modification of floor space available in each pen. For context an average commercial dairy cow in a standing position would occupy 1.8–2 m^2^. In this trial, total space allowance was 9 m^2^ per cow, including bedded area (cubicle lying area), for the control group and 14 m^2^ per cow for the ‘high space’ (intervention) group; this included all areas available to the cows, passageways, bedded areas and feeding areas. Living space, a bespoke previously defined area of the space within dairy cow accommodation which indicates space greater than that considered a baseline requirement^34^, was 3.0 m^2^ for the control group and 6.5 m^2^ for the high space group. These values aligned with the maximum and mean total space and living space measurements recently reported for British dairy farms in 2020^34^.

The remainder of the facility set-up was identical between groups and included one milking robot (Lely Astronaut A4), 39 deep sand freestalls, a robotic brush for environmental enrichment^97^ and two water troughs (183 × 61 cm each). The non-bedded floor area of the pens was slatted concrete which was scraped by robots every 2 h. The feed-face (32.4 m) was located in the centre of the shed for both pens with access through head yokes. Water-filled plastic barriers designed for traffic management^98^ and gates were used to maintain spatial dimensions in each pen.

Environmental conditions included artificial lighting from the Lely Light 4 Cows system^99^. Temperature and humidity were monitored via four sensor locations (ALTA 900 MHz Industrial Humidity Sensors with Probes; ± 3% under normal conditions: 10%–90% RH, optimal battery operating temperature: + 10 °C to + 50 °C). To ensure environmental conditions remained even throughout the research facility, a variable ventilation modular side curtain system (Galebreaker Agri, VVS®) was used in both pens.

### Cow selection and eligibility criteria

The experimental unit was a single cow. Sample size calculations were based on repeated measures models with a power set at 0.8 and a significance probability at 0.05. Separate power analyses were conducted for each experimental outcome to ensure the number of animals was minimised but appropriate, as required by legislation^100^. The most limiting outcome variable was milk production based on repeated measurements over a minimum of 14 occasions. For milk volume (average daily cow volume of approximately 33 kg /d), based on a detectable difference between treatment groups of 0.5 kg/d, a standard deviation between days within cow (corrected for days in milk and parity, based on current herd data) of 0.5 kg/d, a standard deviation between cows (corrected for days in milk and parity, based on current data) of 2 kg/d, a minimum group size of 30 cows per group over the 12 month period was required. To maintain a minimum group size of 30 cows at all times, a starting sample size of 35 cows per group was used. Therefore, seventy Holstein female dairy cows were present in the trial at all times, 35 in each treatment group. Trial design and the normal dairy cow production cycle led to animals exiting the trial at the end of a lactation (‘dry off’) and being replaced with an early lactation cow, a total of 134 unique animals were used throughout the 364-day study (16 cows were used during two different lactations, which was accounted for statistically).

Cows were continuously enrolled into the study in pairs, matched by parity and days in milk (DIM: ± 3 days for cows ≤ 2nd parity, ± 5 days for cows ≥ 3rd parity) and, to ensure equality between groups was maintained, were also removed and replaced as pairs. All cows in the research herd at the University of Nottingham (n = 300) were eligible for initial selection but were excluded from selection if they were (or had been within the last 4 weeks) clinically unwell (received a clinical treatment), had undergone a surgical procedure in their current lactation or if they were greater than 270DIM. Stratified (by parity and days in milk), random sampling was used to select and allocate cows to each treatment group, to ensure overall parity and DIM were equivalent between groups. The parity structure in each group at commencement of trial was: parity 1 = 20, parity 2 = 9, parity 3 = 4, parity 4 = 0 and parity 5 = 2 animals. Randomisation was conducted using the ‘RAND()’ function in Microsoft Excel^101^ to allocate a cow in each pair entering the trial to be randomly assigned into a trial group with the partner entering the other group. All random allocation, enrolment and assignment was undertaken by a single researcher (JT). These decisions were based solely on a report from the farm software detailing: cow identification number, expected/ actual calving date, days in milk and pregnancy status. To ensure cows were in good health all received a veterinary clinical examination on the day of enrolment and were continued to be monitored through the remainder on the trial by a veterinarian.

Cow pairs were removed when the first of the pair reached the time for dry off (± 14 days from 60 days prior to the next calving; n = 36 pairs) or due to ill health (n = 4; 1 × severe lameness, 3 × non-responsive to treatment for metabolic disease) and replaced with a matched pair of recently calved cows (< 14DIM), immediately if dried off or within 3 days if due to a health issue. The numbers of cow-pairs used in total during the 364-day study were 78 (38 of 1st parity, 22 of 2nd parity cows, 8 of 3rd parity cows, 4 of 4th parity cows and 3 of 5th parity). Further details are provided in Fig. 5.

### Routine management of cows

Daily and weekly management routines were identical for both groups of cows and in brief were as follows. The daily feeding routine consisted of a single fresh ad libitum partial mixed ration (based on grass, maize and whole-crop silage) fed in the morning, with feed ‘pushed up’ (physically moved back towards the feedface) every 2 h during the day (8am-8 pm). Concentrates were fed at a rate of 0.45 kg/L above an M + level of 30L for multiparous cows and 26L for primiparous cows via the milking robot. The free stall bedding comprised deep sand which was raked twice each day to remove soiling.

### Cross-over design

Despite the proximity of the research groups (Fig. 5) and matching of all housing infrastructure except living space, a cross over design was implemented to account for unknown variability in environmental conditions due to pen location within the facility. A 364-day period provided a year-long trial which could be split equally into four even section to allow for pen cross-over to account for environmental conditions. Every 91 days the high space and control groups were changed to the contralateral pen. Therefore, cows allocated to either the high-space or control space groups remained within their respective groups but were sequentially swapped to different sides of the building.

### Study outcomes

Primary outcomes measures were assessed as the differences between the two trial groups for production, reproduction and behaviour as follows:

i. Production: 305-day lactation milk yield.
ii. Reproduction: Time taken to conception after calving.
iii. Behaviour: Daily time budgets (times spent in different locations within the pen).

Secondary outcomes measures were:

- Production
  i. Daily and lactation weight of milk solids butterfat.
  ii. Daily and lactation rumination times.
  iii. Bodyweights.
  iv. Concentrate feed intake.
- Reproductive
  i. The time to return to normal oestrus cyclicity post calving.
  ii. The time to return to a normal uterine environment post calving.
  iii. The proportion of oestrus cycles that occur with normal hormonal dynamics and normal inter-oestrus intervals.
  iv. Effect on and of transition cow indicators such as NEFAs and BHBs.

### Data collection

Experimental outcomes: Data were collected in the three principal areas described above; animal production, reproductive performance^54^ and behaviour. With data collected under secondary outcomes measures used to investigate physiological underpinning and interpret the primary outcome measures. There were no complications observed due to ASPA related procedures. Full details of these data collection methods are provided in later sections.

Weekly routines for data collection followed a consistent pattern throughout the trial with trial data collection procedures being conducted on Thursdays. These procedures comprised a veterinary fertility visit, ultrasound scanning and blood sampling.

Blinding: A trained, licensed researcher, JT or MG, performed all sample-taking procedures for the duration of the trial. The researchers could not be blinded to trial groups whilst sampling cows. Most of the outcome measurements were made by sensor or laboratory equipment, thus free from inherent bias. To ensure blinding during the data analysis phase, identifiers on all raw data were artificially recoded by an independent operator, such that all analyses were conducted without knowledge of cow identification or group membership. Once analysis was completed the data were decoded to obtain the trial group results.

Statistical Analysis Software: All analyses were conducted using R statistical software, version R-3.5.2^102^. The following packages were used: Tidyverse^103^, gridExtra^104^, lme4^105^, minpack.lm^106^, influence.ME^107^, survival^108^, survminer^109^.

### Production data and analysis

Data collection and collation: Daily milk volume and rumination data for all cows were obtained from the on-farm Lely Time 4 Cows (T4C) software system^110^. Data were excluded from days when cows were moved between pens (3 cross-over days) because cows were disrupted due to readjustment of the environment and movement of all cows within the environment.

Statistical Analysis: To assess raw data for completeness and visualise data patterns, initial graphical assessments were undertaken. Evaluations of production data were limited to between 1 and 305 DIM since most cows completed a lactation within this time frame. Initial statistical analysis was conducted using a mixed effect model but the fit of these models was poor. Therefore, a two-step analysis was under-taken as described below.

Firstly, non-linear models were constructed to fit the cow lactation curves, using the R package minpack.lm^106^, as described by Ehrlich^47^. The non-linear models took the form (Eq. 1):

1 $$V_{ij} = a\left( {1 - \left( {\frac{{e^{{\left( {\frac{{c - M_{ij} }}{b}} \right)}} }}{2}} \right)} \right)\left( {e^{{ - d*M_{ij} }} } \right)$$

where subscripts *i* and *j* denoted the *i*th observation for the *j*th cow respectively, V was the 7-day rolling milk volume in litres, M the corresponding days in milk (i.e. days after calving) for each cow at each daily recording. Model parameters are depicted as; a = scale; b = ramp; c = offset; d = decay. Model parameters a, b, c and d were initially set at a = 49, b = 27, c = 4, d = 0.00206 as suggested by Ehrlich^47^ and parameter estimates derived for a, b, c and d using the Levenberg–Marquardt non-linear least-squares algorithm^106^.

Using the optimised final non-linear model, residuals were calculated (observed cow daily milk volumes minus model fitted daily milk yield) and residual values were subsequently used as the outcome in a mixed effects model that accounted for data correlations (repeated measures of milk yield over time within cow). Therefore, the model residuals captured the non-linearity of milk yield during lactation and represented a deviation for each cow from an average lactation curve.

Mixed effects models were built in R using the lme4 package^111^. Covariate selection was conducted using a forward step-wise method, and predictor variables retained when *P* < 0.05. Predictor variables included trial-parity group (primiparous-high space; primiparous- control space; multiparous- high space; multiparous-control space) as binary indicator variables and days in milk as a continuous variable. Restructuring of the variables to link intervention group, parity and days in milk was undertaken since this improved fit and clarity of inference of the models. The final model took the form (Eq. 2):

$$O_{ij} = a + \beta_{1} F_{ij} DIM_{ij} + \beta_{2} G_{ij} DIM_{ij} + \beta_{3} H_{ij} DIM_{ij} + u_{j} + e_{ij}$$

2 $$u_{j} \sim N\left( {0, \sigma_{v}^{2} } \right)$$

where subscripts *i* and *j* denoted the *i*th residual milk value of the *j*th cow respectively. $O_{ij}$ was the non-linear model residual (milk deviation) for the *i*th observation of the *j*th cow, $a$ was the model intercept, DIM_ij_ was the day in milk at the *i*th milk yield of the *j*th cow, $F_{ij}$ was a categorical variable to represent primiparous cows in high space trial group, $G_{ij}$ a categorical variable to represent multiparous cows in control space trial group, $H_{ij}$ a categorical variable to represent multiparous cows in high space trial group,$\beta_{1 - 3}$ were fixed effect coefficients, u_j_ a random effect to account for residual variation between cow-parity (assumed to be normally distributed with mean = 0 and variance = σ^2^_v_), e_ij_ was the residual model error.

Model fit was assessed by evaluating covariate influence and delta betas^107^. Q-Qplots were used to visualise and assess the residuals of both the non-linear and mixed effect models. Predictions were generated from both non-linear and mixed effects models to visualise and evaluate results. In addition, for each trial-parity group, a total predicted 305-day milk volume was computed.

### Milk constituents

A monthly milk sample from each cow was analysed for milk protein and fat using an accredited external laboratory (QMMS Ltd, Somerset, UK). A value for the total kilograms of protein and fat produced per cow per day was calculated using the formula: total solid % × 1.03 (milk density) x daily milk volume^112^. Samples where a reading for fat and protein were zero were omitted from the dataset as missing (40 out of 828 samples). A mixed effects model was used to evaluate the effect of treatment group on total mass of milk constituents (kg). Predictor variables tested included trial group (high space vs control), DIM (continuous variable), DIM^2^, DIM^3^ and DIM^4^. Interactions between group and DIM polynomials were investigated and retained when *P* < 0.05. A random effect was included for cow-lactation and model fit was assessed as described above for Eq. (2). The final model took the form (Eq. 3):

$$S_{ij} = a + \beta_{1} G_{j} DIM_{ij} + \beta_{2} G_{j} DIM_{ij}^{2} + \beta_{3} G_{j} DIM_{ij}^{3} + \beta_{2} G_{j} DIM_{ij}^{4} + u_{j} + e_{ij}$$

3 $$u_{j} \sim N\left( {0, \sigma_{v}^{2} } \right)$$

where subscripts *i* and *j* denoted the *i*th data point of the *j*th cow respectively. $S_{ij}$ was the monthly milk solid result for the *i*th observation of the *j*th cow, $a$ was the model intercept; $DIM$ was the day in milk at the *i*th observation for the *j*th cow, $DIM$ was included as polynomial functions to power 2–4, $G_{j}$ was the trial group for the *j*th cow (categorical: high vs control space), β_1–4_ were the vector of coefficients for $G_{j} DIM_{ij}^{1 - 4}$, u_j_ was a random effect to account for residual variation between cow-parity (assumed to be normally distributed with mean = 0 and variance = $\sigma_{v}^{2}$), $e_{ij}$ was the residual model error.

Total milk constituents produced per cow-lactation were calculated using model predictions for 1–305 DIM. Patterns of predicted constituents were assessed graphically to allow a visual comparison between trial groups.

### Rumination

Rumination data were collected from all cows throughout the study using sound monitor microphones in Lely neck collars^113,114^. A mixed effects model was used to evaluate rumination times between trial group. As for Eq. (2), restructuring of the variables to link intervention group, parity and days in milk was undertaken to improve fit and clarity of model inference. The final model specified as (Eq. 4):

$$R_{ij} = a + \beta_{1} F_{j} DIM_{ij} + \beta_{2} G_{j} DIM_{ij} + \beta_{3} H_{j} DIM_{ij} + u_{j} + e_{ij}$$

4 $$u_{j} \sim N\left( {0, \sigma_{v}^{2} } \right)$$

where subscripts *i* and *j* denoted the *i*th data point of the *j*th cow respectively. $R_{ij}$ = daily rumination time in minutes for the *i*th observation of the *j*th cow, $a$ was the model intercept, $DIM_{ij}$ was the day in milk of the ith observation of the jth cow, $F_{j}$ was a categorical variable to represent primiparous cows in high space trial group, $G_{j}$ a categorical variable to represent multiparous cows in control space trial group, $H_{j}$ a categorical variable to represent multiparous cows in high space trial group, $\beta_{1 - 3}$ were fixed effect coefficients, u_j_ a random effect to account for residual variation between cow-parity (assumed to be normally distributed with mean = 0 and variance = σ^2^_v_), e_ij_ was the residual model error.

Using final model parameters, model predictions were made to estimate mean daily rumination times for the period of 1–305 days in milk, for each parity-trial group.

### Reproduction data and analysis

#### Service and conception data

To ensure equality between groups, cow fertility management was based on automated detection of oestrus behaviour; artificial inseminations were exclusively performed when indicated by the automated alerts derived from the Lely T4C software. Pregnancy diagnosis was conducted weekly by a veterinarian with cows checked between 32–39 days post insemination. Decisions for non-pregnant animals were based entirely on a pre-defined standard operating procedure to ensure no differences in reproductive management occurred between groups.

Conception Time-to-Event analysis: Time-to-Event (survival) analysis was undertaken to evaluate and compare the time from calving to conception between treatment groups. Cows were included in this analysis from the point of calving or at the start of the study (if non-pregnant) and the matching of cows at selection meant equality between groups in terms of cow parity and days in milk. Cows that did not conceive were right-censored; time to censoring was calculated as the days from calving to either trial exit or trial end.

A Kaplan–Meier plot was used to visualise survival curves and a Cox proportional hazards models built to estimate the impact of treatment group on time to conception. The proportion of time spent in each side of the building and the amount of time spent in each pen prior to conception were tested as potential confounders in the survival analysis but was found not to influence the final parameter estimates of the model. The Cox proportional model was specified as (Eq. 5):

5 $$h\left( t \right) = h_{0} \left( t \right) \times \exp \left( {\beta_{1} x_{1} \ldots \beta_{p} x_{p} } \right)$$

where t was the time to conception event, $h\left( t \right)$ the hazard function which was dependent on a baseline hazard $h_{0} \left( t \right)$, and the *p* covariates tested took the form ($x_{1}$…$x_{p}$) with related coefficients $\beta_{1}$… $\beta_{p}$.

The covariates were tested as described above and were retained in the model when *P* < 0.05. Visual assessment of Schoenfeld residuals, log–log curves and delta betas were used to evaluate model fit and assumptions^115,116^.

An additional subset analysis was conducted using only pairs of cows that entered the study when less than 14DIM (9 cow pairs removed from initial analysis). This was carried out to check that the nine pairs of cows that commenced the study part way through lactation were not causing bias within the results. The analyses were repeated as above but since no material difference in results was observed, the complete dataset was used for final inference.

Conception Percentage: Data for cow pairs where at least one cow was pregnant prior to study enrolment were removed from analysis (n = 48 cow pairs eligible). The number of artificial inseminations per conception was calculated for each by trial group. This was repeated for the subset of cows that entered the trial < 14DIM (n = 39 eligible pairs).

Inter-Service Intervals (ISI): Inter-service intervals (ISI) were calculated between consecutive serves for all pairs of cows that were not pregnant at entry to study (n = 48 pairs). These intervals were categorised into the following blocks^57^ for analysis : 1-17 days, 18-26 days, 27-35 days, 36-52 days and 52 + days and differences between group evaluated using a Chi-squared test.

#### Measurement of hormonal profiles: progesterone

Data Collection: Milk samples were taken from all cows between 14–84 days in milk on a Monday daytime, Wednesday daytime and Thursday evening/Friday morning for progesterone sample collection. A Lely shuttle milk sampling machine was used according to manufacturer’s recommendations^117^ to automatically collect 25 ml of milk from eligible cows, when they entered into the milking robot. Samples were collected and stored at − 20 °C; analysis of all samples was performed at the end of the study. A commercially available competitive enzyme-linked immunosorbent assay^118^ was used to evaluate progesterone concentration in milk. Samples of 10 µl were analysed in duplicate against standards ranging from 0-50 ng/ml of progesterone. The method used required 200 µl of progesterone-alkaline phosphatase conjugate to be added to the wells, incubated for 2 h and rinsed three times with phosphate-buffered saline (PBS). The plate was left for 1 h after wells were filled with 200 µl of alkaline phosphatase substrate. A colour change was quantified based on absorbance levels using a microplate reader (Optima) when measured at 584 nm. A 4-parameter logistic regression curve was fitted using the absorbance values of the standards and the test sample values were interpolated and quantified from each plate’s standards curve. Data truncation was undertaken when progesterone concentrations were > 20 mg/ml with these readings being recoded to 20 mg/ml based on the range of the standards. The intra and inter-assay coefficients of variation were 13.2 and 13.7%, respectively.

Progesterone Profile Analysis: Individual cow progesterone profiles were assessed visually and categorised using a previously reported methodology^58^. Progesterone profiles were categorised into 3 groups:

i. Normal: First rise in progesterone occurred ≤ 45 days in milk and was followed by normal cyclicity (a short luteal phase < 10 days after the first ovulation was considered typical).
ii. Delayed ovulation (DOV): The first rise in progesterone (> 3 ng/ml) occurred more than 45 days after calving.
iii. Abnormal (combination of three profile definitions):
  - Cessation of cyclicity: The first progesterone rise occurred within the normal period (≤ 45 days) but was followed by a period of progesterone concentrations < 5 ng/ml for > 12 days.
  - Prolonged luteal activity: Progesterone concentration > 5 ng/ml for > 21 days in the absence of AI.
  - Short luteal phase: Progesterone concentration > 5 ng/ml for less than 10 days (excluding the first oestrous cycle).

The numbers of cows with each profile were calculated by trial group and compared by Chi-squared analysis. Time to event analysis was performed as described previously with the conception data but with the addition of these progesterone profile as explanatory categorical covariates.

Commencement of Luteal Activity: The day of commencement of luteal activity (CLA) was defined as the first time after calving that progesterone concentration was > 5 ng/ml for at least 2-time sampling points in a row. If no luteal phase occurred by 84DIM (last sample timepoint), the cow was censored. Commencement of luteal activity between treatment groups was evaluated using survival analysis and a cox-proportional hazards model, as described for the time to pregnancy analysis.

#### Measurement of hormonal profiles: anti-Mullerian hormone (AMH)

Sample Collection: Blood samples of 10 ml were obtained from the coccygeal vein via venepuncture at a single time-point between 22–28 DIM between 9.00–11.00am. For Anti-Mullerian Hormone (AMH) analysis, serum samples were analysed using a bovine specific AMH sandwich ELISA (Ansh Lab, USA, Cat no AL-114) according to manufacturer’s instructions. This ELISA was a quantitative 3-step sandwich type immunoassay. Antibody-antigen-biotin conjugate-SHRP complex bound to the well was detected by enzyme–substrate reaction. Dual wavelength absorbance at 450 nm (primary test filter) measured the degree of enzymatic turnover of the substrate.

Statistical Analysis: Data were collated in Microsoft Excel to calculate concentrations from standards and sample absorbance values. The standards ranged from 16.5 to 2050 pg/ml with samples analysed in duplicate. The intra-assay coefficient of variation (CV) was 5.7%, while the inter-assay CVs were 13% and 1% at 249 pg/ml and 859 pg/ml, respectively. Summary statistics were calculated, and a Kruskal–Wallis rank sum test used to compare values between groups. Additional analyses were conducted to evaluate AMH concentration by cow parity as well as trial group.

#### Ultrasonic assessment of the reproductive tract

Transrectal ultrasound examinations were undertaken at 4 time points for all cows: 14–21, 22–28, 29–35 and 36–42 days after parturition, following a pre-set standard operating procedure. Cows were restrained in head yokes for examination. A researcher (JT or MG) performed the transrectal ultrasound scans using a BCF Easi-scan linear^119^ and measurements consisted of; cross-sectional area measurement of the uterine horns 2-4cms caudal to horn bifurcation, a count of antral follicle (between 2 and 10 mm), a count of antral follicles(> 10 mm) and determination of the presence of a corpus luteum (CL). The distributions of the maximum size of uterine horns and the maximum number of antral follicles per cow at any sampling time points were compared between trial groups using a conventional Students t-test. The identification of a CL prior to 42DIM was analysed using a Chi-squared test to compare the difference between trial groups.

Vaginal examinations were undertaken at 3 time-points: 21–28, 29–35 and 36–42 days after parturition. A “Metricheck” device was used for this examination according to the method described by Pleticha^120^, with scoring for vaginal discharge on a 4 point (0–3) scoring system^120^. A Chi-squared test was used to assess differences in the occurrence of endometritis (present or absent at one or more time points) between trial groups.

#### Transition cow energy balance status

Blood sampling was undertaken at 3 time points for all cows; 7–14, 15–21, 22–28 days after parturition using the venepuncture method described for AMH blood sample collection. Samples were processed, stored and tested within a University laboratory facility (NUVETNA laboratory, Sutton Bonington, UK). Non-esterified fatty acid (NEFA) and beta-hydroxybutyrate (BHB) levels were determined using standardised colorimetric kits (Randox Laboratories, Crumlin, UK) within the RX IMOLA machine (Randox Laboratories, Crumlin, UK) according to manufacturer’s instructions^121^.

Summary statistics were calculated for BHB and NEFA by trial group. The total number of high BHB or NEFA results in each group were assessed; BHBs were classed as high when ≥ 1.2 mmol/l and NEFAs when ≥ 0.8 mmol/l^122^. The effect of potential energy balance on reproductive performance was assessed by removing cow-pairs from the time-to-conception model when at least 1 individual had a minimum of one high NEFA or BHB results. Time-to-conception analysis was repeated using this smaller sub-sample of eligible cow-pairs (n = 24 cow-pairs). A Kaplan–Meier graph and Cox-proportional model were constructed as described above for the time-to-conception analysis.

### Relationships between milk volume and conception: statistical analysis

#### Test for the impact of reproductive performance on milk volume

To test the effect of reproductive status on milk yield, daily milk volume data were combined with reproductive conception data at the cow level. A new variable was defined for each cow on each day of the study, to label the number of days pregnant (DP), using 0’s for days non-pregnant and a continuous integer scale from day of conception until trial exit. The DP variable was recoded as a categorical covariate as follows; 0 (non-pregnant), 1 (1–50 DP), 2 (51–100 DP), 3 (101–150 DP), 4 (151–200 DP), 5 (201–250 DP) and 6 (250 + DP). These were aligned to the cows previous 7-day rolling milk volume data at the corresponding DIM.

Raw data were assessed to visualise milk produced by cows in each trial group dependent on pregnancy status. The methods for the mixed effects model (Eq. 2) were used to test the model to include the new DP variable alongside the final predictor variables, which included DIM (continuous) and trial-parity group (primiparous-high space; primiparous- control space; multiparous- high space; multiparous-control space) as binary indicator variables.

Model fit was assessed, and predictions of lactation curves made for each trial group and parity-specific time to conception outcome (primiparous- high space, primiparous control space, multiparous high space and multiparous control space). For comparison, scenarios where days to conception were set at 80, 120, 130 and 160 days were also explored for both trial groups alongside the observed reproductive outcomes from the days to conception survival analysis. The 305-day milk volume predictions were made for all trial-parity groups and compared.

#### Test for the impact of milk volume on time to conception

Pregnancy status was set as a binary variable (0/1), zeros were assigned for each day in milk a cow was not pregnant. When a cow reached the day of conception this variable was assigned a 1 and all data were removed beyond this DIM row. Data were filtered to only include the 48 cow-parity pairs where both cows were non-pregnant at trial entry, the same population as used in the time to event analysis for days to conception.

A conventional mixed-effects, binomial logistic regression model was used to test whether the probability of pregnancy was associated with the previous 7-day rolling milk volume. The outcome was conception as a binary variable (0/1). Predictor variables included in the model were 7-day rolling milk volume (continuous), ln(DIM) (also tested as polynomial functions to power 4) and trial group (binary factor high space or control group). Cow was fitted as a random effect to account for repeated outcome events within cow. Model fit was evaluated by comparing prediction probabilities to actual observed events for parity 1–4 and by trial group^123^.

### Cow behaviour data and analysis

*Data collection*: A wireless sensor-based location system (Omnisense Cluster Geolocation System, UK)^124,125^ was used to track the movements of a subset of cows for the final 5 months of the study period in real time. The subset of paired cows (n = 16 pairs) comprised those earliest in lactation when the sampling period commenced and were between 9 and 77 days in milk. A location tracking device was fitted to a neck collar on each of the participant cows. The cow sensor was fixed in position on the dorsal aspect of the cow’s neck (to enhance positioning relative to fixed sensors) using a counterweight (0.5 kg). Originally, location data were planned to be obtained for the entire duration of the trial period for all cows but due to technical issues such as connectivity and durability, this was not possible. Therefore, a new hypothesis (with sample size calculation) was developed incorporating to provide behavioural data from this carefully matched subset of cows.

The Omnisense system used a wireless mesh network to determine the relative location of each sensor from known fixed sensor positions. The relative spatial location of the on-cow sensors was determined and data were transmitted to the server remotely using ultra-wideband radiowaves from 3.5 to 6.5 GHz, IEEE802.15.4, to connect to the location server. This provided an accuracy of ± 50 cm at 95% confidence (which improves to ± 20 cm under good conditions). Location data were obtained every 9 s on average (0.11 Hz) over the sampling time period, to give appropriate battery function of ~ 7 days before replacement was necessary. All data were stored and analysed at the end of the trial.

Data analysis: A total of 29.7 million data points were collected. Initial visualisation of all datapoints was undertaken using heatmaps in R^103^. To improve data reliability and data handling capacity, the mean coordinate for each cow in every 1-min period throughout the sampling period was calculated resulting in 3,738,739 data points. Where there was no data for a cow in a 1-min period this data was classed as missing.

The research buildings were divided into key zonal areas which were defined by the underpinning grid of x and y coordinates; cubicles, milking robot, water trough, brush, feed-face, and “other” (movement area- i.e. non-feeding area of feed passageway) as well as the loafing area for the high space group. These were physically measured by the researcher during the trial period to ensure co-ordinates were correctly positioned; the measurements were compared to the geolocation set base co-ordinate (0,0) and the corresponding co-ordinate of the zone was calculated. Co-ordinates were assigned to each zone. Using the R “fuzzyjoin” package^126^, cow location co-ordinates were allocated to the corresponding key zonal area for each observed datapoint. For example, the edge of the ‘cubicle zones’ were defined exactly at the front of the lying area to increase specificity with regards to lying behaviours. Pilot work prior to commencement of the trial identified that when cows were in the defined ‘cubicle’ or ‘feeding’ zones they were undertaking lying and feeding behaviours respectively. However, the location of cows in specific area zones are reported rather than the behaviours which they are exhibiting to account for the lack of granularity in this dataset.

Direct comparisons of daily time budgets were made between trial groups. Time budgets could be impacted by stage of lactation due to a cow’s metabolic demands but the matched nature of this trial should account and control for these differences over time when comparing the two trial groups. Tests were undertaken to check consistency of time budgets on different sub-datasets; all data (n = 3,738,739; cow pairs = 16), exact match minute data for both cows in a pair (n = 2,410,756; cow pairs = 16), and matched pairs with full-matched coverage (n = 1,768,094; cow pairs = 5). Key behavioural activities evaluated were time spent in designated areas such as loafing areas, cubicles and environmental enrichment use.

To assess the uncertainty associated with the time budget results, bootstrapping of the data was undertaken using two methods. The first bootstrap method used was sampling with replacement within each cow’s data to ensure all cows remained equally represented within the bootstrapped data. The second method was to sample with replacement from all data rows. Re-sampling was undertaken 500 times per trial group for both methods and 95% confidence intervals were calculated for the mean time spent in each zone by cows in each trial group.

## Data Availability

The data analysed to support the results of this randomised controlled trial are available on request from the corresponding author [MG]. Data are not publicly available due to them containing information with the potential for compromising research participant privacy.
